# Progress in Surgery

**Published:** 1897-08-28

**Authors:** 


### PROGRESS IN SURGERY.

### ORTHOPÆDIC PROGRESS.

(*Continued from page* 358.)

**Achillodynia.**—Romme[11] remarks that this affection is characterised by the fact that standing and walking become impossible because of the severe pain they cause. This pain disappears entirely when the foot is elevated and at rest. It is located exactly at the insertion of the tendo Achillis. There is often slight swelling at this spot. The affection is undoubtedly due to inflammation of the bursa, between the tendon and the posterior surface of the os calcis. In the interior of the bursal cavity there are almost constantly found fatty fringes coming from the adipose periarticular tissue. Usually, the walls of the bursa are much thickened, and often there is a hypertrophic periostitis with fatty degeneration of the hypertrophied layers. Sometimes the walls of the bursa are almost cartilaginous. The treatment is very simple in acute cases; rest, pressure, and evaporating lotions are sufficient to give relief. In chronic cases massage and pressure by means of a moist sponge held in place by wet bandages usually suffice to bring about a cure. If the symptoms persist, incision with drainage or extirpation of the bursa is advisable. Weir[12] gives a case of this affection treated by operation. The patient, Jacob M——, aged fourteen years, gave no history of tuberculosis in the family. He entered the New York Hospital in October, 1896. Three months previously he sprained his heel by getting his foot caught in a railing. After a week, as he continued limping, he was kept in the house and liniments were applied. He remained a good deal of time in bed with pain in the heel and ankle. On examination the foot was found strongly extended, with fulness and much tenderness on either side of the tendo Achillis. Active motion was prevented by pain. Passive motion was also painful. No pain was produced by crowding together the joint surfaces. October 10th, under ether, a curved incision was made from below the outer malleolus and running upward along the tendo Achillis. The bursa underlying this tendon was opened. It contained about two drachms of sanguino-purulent material, and its walls were found thickened and much congested. The bursa was dissected out, and the posterior portion of the os calcis, thought to be softened but unchanged in colour, was scooped out to a moderate extent with a Volkmann's spoon. Iodoform powder was rubbed into the tissues, and the wound was sutured with fine silk over a drain. The cavity closed by granulations, and at the time the patient left the hospital, four weeks after the operation, there was still a sinus discharging sero-purulent fluid. No tubercle bacilli were found in the fluids at the operation, nor did the secretions show the characteristic lesions of tuberculosis. April 2nd, 1897, about five months after the operation, he was again seen. The functions of the foot were fully restored. Dr. Weir remarked that achillodynia, in its obstinate form, has but recently been treated by operation. The observations of Rössler, of Vienna, of 225 bursæ underlying the tendo Achillis have shown frequent lesions, not only those belonging to acute inflammations, but changes, such as bone hypertrophies, calcific plates, tuberculosis, and even sarcoma, all of which can be attacked only surgically. Wiesenger, of Hamburg, has published four cases of tuberculosis achillo-bursitis treated by extirpation of the bursa and adjacent bone, and also one case of sarcoma of the same bursa. He suggests, if necessary, to cut across the tendon to expose the bursa, and subsequently to suture it together again.

**Morton's Disease, or Metatarsalgia.**—Several papers have appeared on this subject recently. Tubby[13] has written extensively on this affection, and a most interesting and orginal article is also contributed by Mr. Robert Jones[14] under the title of "Plantar Neuralgia." After a brief review of the literature of the subject, the writer treats of the arches of the foot, and says that they are not separate, but they are integral portions of the whole; that is, if the longitudinal arch is flattened the transverse arch is too. He has also been at pains to demonstrate the relative position of the heads of the metatarsal bones by frozen sections, this having been done in view of the explanation advanced by Morton as to the cause of the disease. Mr. Robert Jones also draws attention to the fact that a communication exists between the fourth branch of the internal plantar nerve and the innermost branch of the external plantar nerve. Later on in the article he shows that this communication is of great importance in the production of the pain characteristic of this affection. He divides metatarsalgia into three degrees. In the first, those cases are placed in which pain is occasionally felt along the metatarso-phalangeal joints during the performance of certain acts, but is immediately recovered from on desisting from such acts, and acute pain is not produced by firmly holding the fourth metatarso-phalangeal articulation between the thumb and forefinger. In the second degree are those cases where characteristic symptoms quickly follow early attempts at walking after an injury due probably to a breaking down of the transverse arch. The third, or severe variety, comprises those cases where symptoms appear idiopathically or remotely after injury, are persistent in character, do not yield to mechanical measures, and to all effect cripple the patient; and severe pain is felt on firmly holding the fourth metatarso-phalangeal articulation between the finger and thumb. Numerous cases illustrative of these degrees are quoted. The symptoms are as follows: Pain over the metatarso-phalangeal region on walking only slightly relieved by mechanical contrivances; pain on pinching the fourth metatarso-phalangeal articulation; relief of pain on circumferential pressure around the base of the toes, assisted still further by repeatedly flexing the toes; sensation of walking on something hot; and there are generally present flattened arches of the foot. This condition of flattening of the foot is by no means seen in all cases. In some instances there is found to be an increased arch of the foot. From an analysis of symptoms in 17 cases quoted in Mr. Robert Jones's paper it is noted that there was a history of injury in 10 cases; that the third joint was affected as well as the fourth in 3 cases; that lateral pressure gave relief in 14; that reference was made to subluxation or reduction with a click in 7; that there was flattening of both longitudinal and transverse arches in 12; that 10 patients were women, and 7 men; that 3 described sensations as if standing on something hot; that the ages ranged from 24 to 58, 10 being from 30 to 40. In one case symptoms were induced by a small fibroma of the plantar fascia. Symptoms of gout, rheumatism, or hysteria were only found in two cases, and flexion of the toes in the majority of cases was productive of relief. With reference to the etiology, Robert Jones, in contrast to Morton—who believes that the affection is due to pinching of the digital nerves between the heads of the metatarsal bones—is of opinion that the pain arises from stretching or pressure upon, rather than nipping of the nerves, and this opinion is borne out by the following points, namely, the depression of the anterior arch and the ease experienced by circular compression, and the pain due to local pressure with finger and thumb over the fourth metatarsal articulation. With reference to treatment, the following points are advocated: (1) To abstain from any action which produces the pain; (2) to increase the depth of the inner aspect of the heel of the boot in order to produce slight eversion of the foot; (3) to wear thick soles with well-fitted insteps, and roomy round the heads of the metatarsal bones; (4) that the soles should be at least one-fourth of an inch thicker a little behind the heads of the metatarsal bones. The foregoing measures are sufficient to ensure relief in mild cases, but in the second stage certain other details should be attended to. It is well to have a thick bar placed about half an inch behind the heads of the metatarsal bones, and to fix around the instep a band of non-irritating plaster. Massage of the foot, with contrast baths of hot and cold water, and elevation of the foot of the bed at night time, are also advocated. In the severest description of cases nothing short of an operation brings relief. Three operations are in vogue—exsection of the head of the metatarsal bone, excision of the metatarso-phalangeal joint, and amputation of the metatarsal head and toe. Other measures have been tried, but they are not reliable. Such measure are the actual cautery, a heated needle over the point to destroy the nerve, hypodermic injection of carbolic acid, and exsection of the digital plantar nerve. The most reliable form of operative treatment is exsection of the head of a metatarsal bone, generally the fourth.

[11] La TribuneMédicale, No. 14, 1896. [12] New York Med. Record, April 29th, 1897. [13] Lancet, Oct. 31st, 1896, and Clinical Journal, May 26th, 1897. [14] Liverpool Med. Chir. Journal, January, 1897.

### DISEASES OF THE PHARYNX AND LARYNX.

(*Continued from page* 356.)

A case of double abductor paralysis was recently shown by St. Clair Thomson,[9] a case that had been recorded by Sir Morell Mackenzie in 1870,[10] at a time when the paralysis was limited to the right side, and then believed to be due to injury to the pneumogastric trunk. Sir Felix Semon, in discussing the case, pointed out that the late Sir Morell Mackenzie, in his description of this case, had only spoken of paralysis on the right side of the larynx, whilst at present there could be no doubt that there was bilateral—and, indeed, very complete—abductor paralysis. The explanation probably was this: According to Mackenzie's description the pneumogastric nerve had been injured; hence, probably, an ascending neuritis had occurred, the affection had extended from the vagus nucleus to the nucleus of the spinal accessory in the medulla, from the latter through the commissural fibres (which, according to Lockhart Clarke, connected the two accessory nerves) to the left accessory nucleus, and had then travelled downwards the trunk of that nerve, causing abductor paralysis on the left side as well (Sir George Johnson's theory). In view of the fact, however, now communicated by Dr. St. Clair Thomson, that the patient had had syphilis prior to the infliction of the injuries, it was, of course, also possible that all the various nerve lesions met with in his case might be the result of cerebral syphilis.

A unilateral paralysis of the vocal cord, due to rheumatic neuritis, was shown at a recent meeting of the Laryngological Society of London, by Watson Williams.[11] The left abductor was paralysed and the adductor paretic when first seen, but when shown only abductor paresis was present. It was an interesting fact that the adductor recovered first; and Williams recalled another instance of this tendency to the early recovery of the adductor, as compared with the abductor, viz., that of a child, whose right vagus had been divided during an operation on the neck (the cut ends being sutured immediately), leaving only a pure abductor paralysis. Sir Felix Semon had demonstrated the proneness of the abductors to succumb earlier than the adductors; these cases afforded clinical evidence of the converse sequence of events in recovery, viz., that the adductors exhibit a decided tendency to recover sooner than the abductors.

**Toxic Paralysis of the Larynx** is discussed by Heymann, whose extensive *résumé* of over fifty collected papers shows that lead poisoning is responsible for the majority of cases, and the author adds three to the number already published. The author shows that it is the abductor muscles which are mainly affected. In his own cases these alone were paralysed. Poisoning by copper, antimony, phosphorus, and arsenic has been responsible for a small number; and the author has met with three instances of arsenic paralysis, which, after temporary disappearance, recurred on re-exposure to the influence of the poison. Laryngeal palsy has also been attributed to various organic poisons—such as cannabis indica, atropine, morphia, alcohol, &c.

**Disseminated Sclerosis (Sclerose en Plaques).**—Collet[13] records a case of a young man, aged 24, with medullary implication, who, besides showing the special signs of disseminated sclerosis, had interrupted, stuttering speech. Collet found, on laryngoscopic examination, very pronounced oscillations of the vocal cords; when the patient spoke they were agitated by a true tremor.

**Varicellous Laryngitis.**—Roger and Bayeux[14] describe a case of an infant of six months which succumbed to hæmorrhagic varicella. Progressive dyspnœa developed during the last thirty hours of life. Post-mortem, the authors found gangrene of the edge of the epiglottis, a strip of slough on the free borders of the vocal cords, a crateriform erosion on the left vocal cord, and a varicella spot on the mucous membrane of the left pyriform fossa.

**Scarlatinal Laryngitis.**—Knyvett Gordon[15] recently showed a pharynx from a case of scarlet fever, fatal on the ninth day of disease, that had been treated with anti-streptococcic serum. The specimen showed sloughing of both tonsils, and ulceration of the uvula. There was a chain of small ulcers, extending from the tip of the epiglottis to the pyriform fossa on each side; the naso-pharynx had been full of sloughing adenoid tissue. He stated that, in his opinion, the serum was not of much value in cases of scarlet fever where the septic symptoms appeared late, and were due to absorption from the sloughing throat or neck; but he had obtained a strikingly good result when there was septicæmia at the onset, and the serum was given early.

**Angina Epiglottidea Anterior.**—A case of this rare conditionis recorded by Meyjes,[16] of Amsterdam. The patient complained of sore throat, painful deglutition, and choking on attempting to swallow milk or soup; the voice was clear. The illness had begun with a slight cold some weeks before. He was very ill and feverish. The fauces were almost normal, but the anterior surface of the epiglottis was found to be very red, and extraordinarily swollen, while the posterior surface of the epiglottis and the true cords were slightly red. The author states that he ordered absolute rest, aperient water, ice internally and externally, and last, but not least, the use of an iced spray of one-third per cent. watery solution of ichthyol every quarter of an hour. He is acquainted, he says, with no drug that diminishes redness and swelling so quickly, and has used it during the last six years in all cases of acute inflammation. It is free from the slightest irritation, and although the smell is not agreeable, patients soon get accustomed to it, if prescribed for the first few days not stronger than one-fourth to one-third per cent. On the following day the patient was already much better; the pain had lessened, and the fever abated. On examining the larynx he found the epiglottis less swollen and the redness diminished. Scarification—which he intended to perform had the symptoms continued or increased—was then unnecessary, and in about a week only slight infiltration remained at the base of the epiglottis.

This form of œdema laryngis, strictly limited to the epiglottis, especially its anterior surface, has been termed *Angina epiglottidea anterior* by Michel of Cologne ("Centralblatt für Medicinische Wissenschafte," 1878, No. 2), who first described cases of this kind. He found the pharynx almost unaffected, and without laryngoscopy a correct diagnosis could not be made. He described six cases, two associated with swelling of the ary-epiglottic folds, one with hoarseness due to swelling of a vocal cord, and two complicated with angina Ludovici, the inflammation having passed to the subcutaneous tissues. When not detected early, the life of the patient is in great danger, owing to the possible extension of the process. Moritz Schmidt ("Die Krankheiten der Oberen Luftwege," s. 153) describes this disease as often associated with, or preceded by, angina acuta simplex. He thinks that it is always caused by an injury or inflammation of the lingual tonsils (s. 232). In Meyjes' case the illness may have been ushered in by angina, as the patient suffered from such attacks several times a year. It is remarkable, however, that on no previous occasion were signs observed of the epiglottis being involved. [We would draw attention to Sir Felix Semon's views as to the pathological identity of all these septic throat inflammations, and express our belief that the so-called "angina epiglottidea anterior" is simply a localised septic infection of the epiglottis, which, when occurring elsewhere, is termed "angina Ludovici," "septic pharyngitis," &c.—Ed.]

[9] Proc. Laryng. Soc. of Lond. [10] Brit. Med. Journ., Dec. 24, 1870. [11] Proc. Laryng. Soc. of London, Jan., 1897. [12] Arch. Intern. de Laryng. Ot. et Rhin., No. 6, IX., 1876; Journ. of Laryng., Mai., 1897. [13] Lyon. Med., Jan. 24, 1897; Journ. of Laryng., June, 1897. [14] Press Med., Ap. 10, 1897; Journ. of Laryng., May, 1897. [15] Proc. Laryng. Soc. of London, 1897. [16] Journ. of Laryng., March, 1897.

